# Upregulation of B7-H4 promotes tumor progression of intrahepatic cholangiocarcinoma

**DOI:** 10.1038/s41419-017-0015-6

**Published:** 2017-12-13

**Authors:** Nan Xie, Jia-Bin Cai, Lu Zhang, Peng-Fei Zhang, Ying-Hao Shen, Xuan Yang, Jia-Cheng Lu, Dong-Mei Gao, Qiang Kang, Li-Xin Liu, Chi Zhang, Xiao-Yong Huang, Hao Zou, Xin-Yu Zhang, Zheng-Ji Song, Hai-Xiang Sun, Bi-Mang Fu, Ai-Wu Ke, Guo-Ming Shi

**Affiliations:** 1 Key Laboratory of Carcinogenesis and Cancer Invasion of Chinese Ministry, Department of Liver Surgery and Liver Transplant of Zhongshan Hospital, Liver Cancer Institute of Fudan University, Fudan University, Shanghai, 200032 China; 2 Department of Hepatopancreatobiliary Surgery, The Second Affiliated Hospital of Kunming Medical University, Kunming, Yunnan 650101 China; 3 Department of Gastroenterology, The First People’s Hospital of Yunnan Province, 157 Jin Bi Road, Kunming, Yunnan 650032 China

## Abstract

Recent reports show that B7-H4 is highly expressed in a variety of tumor cells, functions as a negative regulator of T cells and then promotes tumor progression. However, its expression and role in intrahepatic cholangiocarcinoma (ICC) remain unclear. In present study, B7-H4 expression in ICC and peritumoral tissues was determined at the level of mRNA and protein, and its bioactivity in ICC cells was studied after modification of B7-H4 expression. Then, the mechanism related to tumor progression induced by B7-H4 expression in ICC cells was explored. Finally, clinical significance of B7-H4 expression in ICC patients was further analyzed. The results showed that B7-H4 expression in ICC was much higher than that in peritumoral tissues at the level of both mRNA and protein. The high level of B7-H4 in ICC cells induced epithelial-to-mesenchymal transitions and promoted invasion and metastasis of tumor cells through activation of ERK1/2 signaling. The elevated B7-H4 expression was associated with the downregulated Bax, upregulated Bcl-2 expression, and activation of caspase-3. Clinically, high B7-H4 expression in tumor samples was significantly related to malignant phenotype, such as lymph node metastasis, high tumor stage, and poor differentiation. ICC patients with high expression of B7-H4 had shorter overall survival (OS) and disease-free survival. Moreover, the B7-H4 expression was an independent prognostic factor for predicting OS and tumor recurrence of ICC patients after operation. In conclusion, high expression of B7-H4 promotes tumor progression of ICC and may be a novel therapeutic target for ICC patients.

Intrahepatic cholangiocarcinoma (ICC) arising from the epithelial cell of intrahepatic bile duct ranks as the second most common primary liver cancer and is characterized by early metastasis and dismal prognosis with < 5% of whole cohorts for 5-year survival^1^. Epidemiological data show that the incidence, prevalence, and mortality of ICC is steadily increasing in Eastern Asia and many Western countries during the last two decades^2,3^. Now, curative resection is still the first choice for treatment of ICC. Unfortunately, it is only suitable for a small fraction of ICC patients. Gemcitabine-based chemotherapy could prolong overall survival (OS) for advanced ICC patients, but the benefit was limited with ;< 1-year survival for those patients^4,5^. Thus, a better understanding of the molecular mechanisms of ICC is necessary to define novel targets for early diagnosis and therapeutic intervention.

B7-H4 (also named VTCN1) belongs to a new member of the B7 family. Recent studies showed that B7-H4 could promote the cell proliferation and cytokine secretion of T cells, regulate cell cycle, and suppress the growth of neutrophil progenitors, thereby enabling tumors to avoid immune detection^6,7^. In general, B7-H4 is the absence in normal human tissues, excluding epithelial cells of lung^8^, kidney^9^, and pancreas^10,11^. Recently, several studies reported that B7-H4 was found to be highly expressed in several cancers including lung^8^, ovarian^12^, breast^13^, prostate^14^, renal^9^, gastric^15^, esophageal cancers^16^, and pancreatic ductal adenocarcinoma^17^. Moreover, high level expression of B7-H4 in tumor tissues was regarded as a potential unfavourable prognostic indicator for these patients^8–10,13–15^. However, the role and mechanism of B7-H4 in ICC remain elusive.

In this study, our aim is to investigate the expression of B7-H4 and its clinical significance in ICC. The role and molecular mechanism of B7-H4 in ICC were also determined.

## Results

### B7-H4 was highly expressed in ICC tissues

**Fig. 1:**
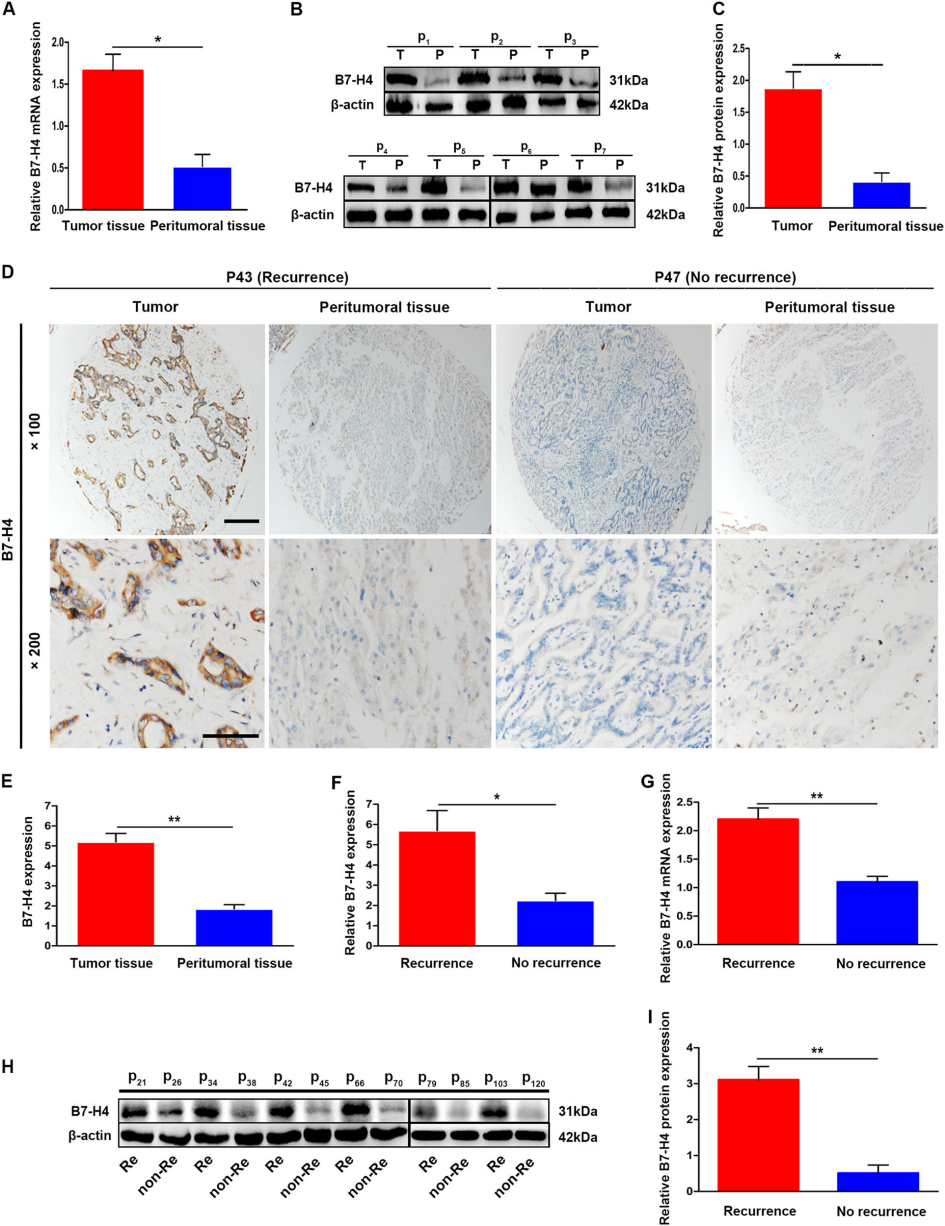
High expression of B7-H4 in tumor tissues of ICC patients. **a** qRT-PCR showed that the expression of B7-H4 mRNA in tumor tissues was higher than that in correspondingly peritumoral tissues (*p < 0.05). **b** Representative image showed expression of B7-H4 protein in tumor samples and peritumoral tissues from 35 ICC patients by western blotting. **c** Semi-quantitative analysis showed that the expression of B7-H4 protein in tumors tissues was higher than that in peritumoral tissues(*p < 0.05). **d** Representative image showed the expression of B7-H4 protein in tumor samples and peritumoral tissues from 140 ICC patients by immunohistochemical staining. **e** Semi-quantitaive analysis for IHC staining showed that B7-H4 protein expression in tumor samples was higher than that in peritumoral tissues (**p < 0.01). **f** The expression of B7-H4 in tumor tissues from ICC patients with early recurrence after operation was higher than that in patients without recurrence (*p < 0.05). **g** qRT-PCR from 35 ICC patients showed that the expression of B7-H4 mRNA in tumor tissues of ICC patients with early recurrence was much higher than that of patients without recurrence (***p < 0.01). **h** Representative image showed expression of B7-H4 protein in 35 randomly selected ICC pateints by western blotting. **i** Semi-quantitaive analysis for western blotting showed that the expression of B7-H4 in tumor tissues from ICC patients with early recurrence was higher than that in patients without recurrence (**p < 0.01)

Here we first detected the B7-H4 expression in tumor tissues and their corresponding adjacent non-tumor tissues from 35 ICC patients by quantitative real-time PCR (qRT-PCR) and western blotting. As shown in Fig. 1a–c, the B7-H4 expression in tumor tissues was much higher than that in adjacent non-tumor tissues (messenger RNA, 1.65 ± 0.21 vs. 0.51 ± 0.13 and protein, 1.81 ± 0.42 vs. 0.43 ± 0.11, *p* < 0.05, respectively). We further used immunohistochemistry (IHC) to investigate the expression and location of B7-H4 in tumor samples from 140 ICC patients. The results showed that positive staining for B7-H4 protein was mainly localized in the cell membrane of tumor cells and its intensity in tumor tissues was evidently stronger than that in corresponding adjacent non-tumor tissues (Fig. 1d, e). Interestingly, semi-quantitative analysis for IHC staining showed that tumor samples from ICC patients with early recurrence (< 2 years after operation) had stronger staining of B7-H4 than those in patients without early recurrence (Fig. 1d, f).

We also analyze the correlation between B7-H4 expression in tumor samples using western blotting and qRT-PCR, and early recurrence in 35 ICC patients. As shown in Fig. 1g–i, B7-H4 expression in tumor samples from ICC patients with early recurrence was much higher than those in ICC patients without early recurrence (mRNA, 2.08 ± 0.13 vs. 1.22 ± 0.08 and protein, 3.11 ± 0.26 vs. 0.51 ± 0.16, *p* < 0.01, respectively). The above results indicate that B7-H4 expression probably promote the tumor progression of ICC.

### High B7-H4 expression positively correlated with poor prognosis of ICC patients

**Fig. 2:**
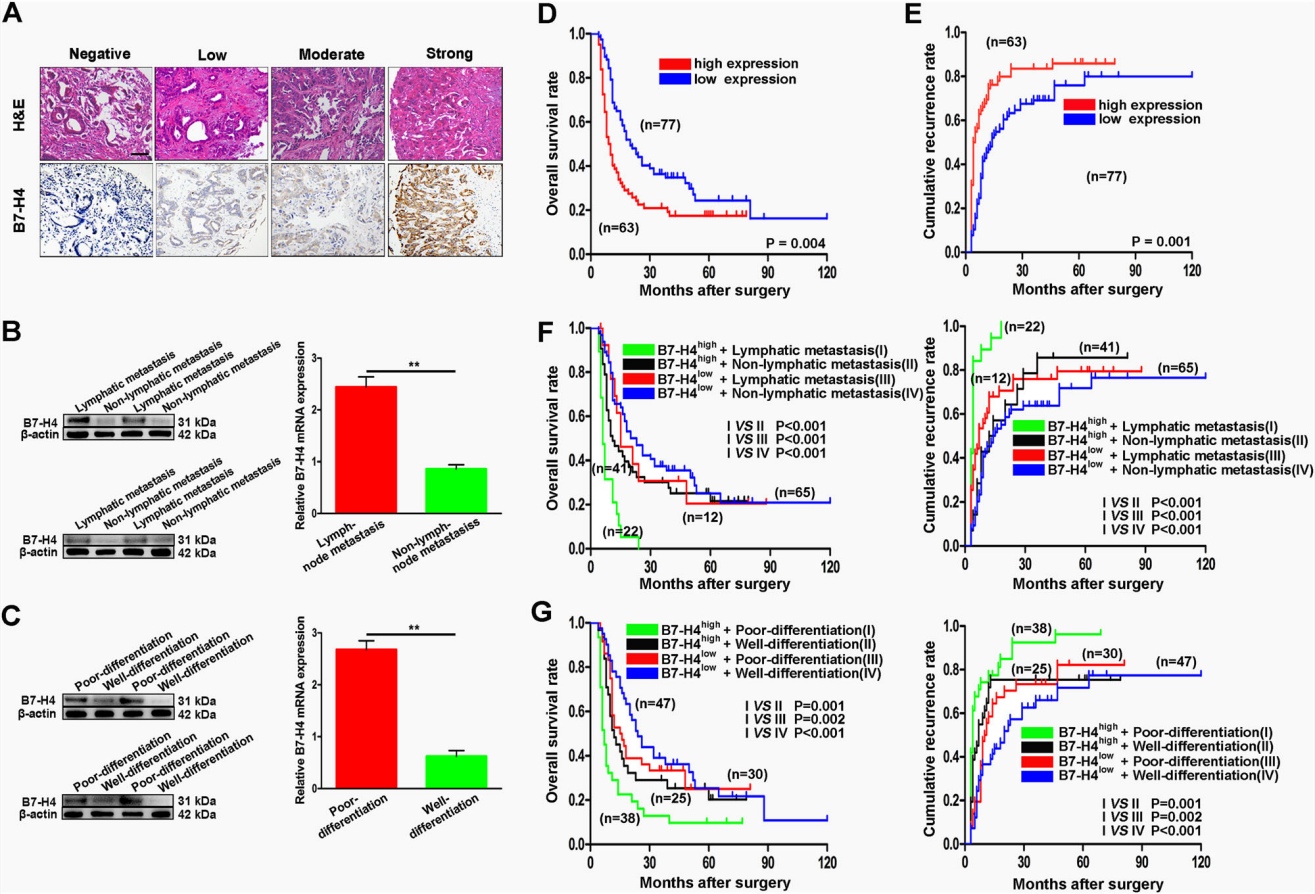
Prognostic significance of B7-H4 for ICC patients. **a** Representative image showed different expression of B7-H4 in different ICC tissues by IHC staining. **b** Semi-quantitaive analysis for western blot and qRT-PCR showed that ICC patients with lymph node metastasis had higher expression of B7-H4 than those patients without lymph node metastasis(**p < 0.01). **c** Semi-quantitaive analysis for western blotting and qRT-PCR showed that ICC patients with poor tumor differentiation had higher expression of B7-H4 than those patients with well tumor differentiation (*p < 0.01). **d** Survival analysis showed that ICC patients with high B7-H4 expression had a shorter overall survival compared with those patients with low B7-H4 expression (**p = 0.004). **e** Survival analysis showed that ICC Patients with high B7-H4 expression had higher cumulative recurrence rate compared with those patients with low B7-H4 expression (**p = 0.001). **f** Survival analysis showed that ICC Patients with high B7-H4 expression and lymphatic metastasis had the poorest prognosis among four sub-groups (***p < 0.001). **g** Survival analysis showed that ICC patients with high B7-H4 expression and poor differentiation had the poorest prognosis among four sub-groups (**p < 0.01)

Here we further investigated the clinical significance of B7-H4 expression in 140 ICC patients. The cliniopathological features of 140 ICC patients were summarized in Table 1. As shown in Fig. 2a, B7-H4 staining in each tumor tissue was greatly varied. Then, we dichotomized whole cohort into B7-H4^high^ (the combined score for B7-H4 expression >3 points; *n* = 63) and B7-H4^low^ (the combined score for B7-H4 expression ≤3 points; *n* = 77) subgroup, and analyzed the relationship between the level of B7-H4 expression and clinicopathological features of ICC (Table 1). The results showed that high level of B7-H4 expression was significantly related to lymph node metastasis (*p* = 0.008), high TNM stage (*p* = 0.024), and poor tumor differentiation (*p* = 0.012). However, neither of other clinicopathological features, including age, sex, serum AFP, and tumor number, was associated with B7-H4 expression. Then, we compared the relationship between B7-H4 expression and malignant phenotypes. The data from 35 ICC patients used for western blotting and qRT-PCR assay showed that ICC patients with lymph node metastasis had higher expression of B7-H4 compared with those patients without lymph node metastasis (Fig. 2b). Meanwhile, ICC patients with poor tumor differentiation tended to have higher expression of B7-H4 compared with those patients with well tumor differentiation (Fig. 2c). Kaplan–Meier analysis revealed that ICC patients with high levels of B7-H4 had a significantly shorter OS and higher cumulative recurrence rate than those with low levels of B7-H4 (*p* < 0.01, Fig. 2d, e). We further examined the combined role of B7-H4 expression with malignant phenotypes, including lymphatic metastasis and tumor differentiation in OS and recurrence rate. Our results showed that ICC patients with high levels of B7-H4 and lymphatic metastasis had the worst prognosis in term of OS rate and recurrence rate among four subgroups (*p* < 0.001, Fig. 2f). Similarly, ICC patients with high levels of B7-H4 and poor differentiation had the worst prognosis among four subgroups (*p* < 0.01, Fig. 2g).

**Table 1:** Correlation between B7-H4 expression and cliniopathological features in 140 intrahepatic cholangiocarcinoma patients

Variables	B7-H4 staining	*p-*value^a^	
	High	Low	
*Age (years)*			
≥ 53	34	36	0.396
< 53	29	41	
*Sex*			
Male	25	34	0.594
Female	38	43	
*HBsAg*			
Positive	42	45	0. 318
Negative	21	32	
*Liver cirrhosis*			
Yes	22	35	0.207
No	41	42	
*Serum AFP* ^*^ *(ng/ml)*			
< 20	58	64	0.116
≥ 20	5	13	
*Serum ALT (μ/l)*			
≥ 75	10	9	0.472
< 75	53	68	
*Child–Pugh score*			
A	60	74	0.801 ^b^
B	3	3	
*Serum CA19-9* ^*^ *(ng/ml)*			
≥ 37	41	44	0.339
< 37	22	33	
*Microvascular/bile duct invasion*			
Yes	10	13	0.873
No	53	64	
*Tumor size (diameter, cm)*			
≤ 5	53	56	0.106
> 5	10	21	
*Tumor number*			
Multiple	6	5	0.507
Solitary	57	72	
*Tumor differentiation*			
III/IV	38	30	0.012
I/II	25	47	
*Lymphatic metastasis*			
Yes	22	12	0.008
No	41	65	
*TNM stage* ^*^			
III/IV	25	17	0.024
I/II	38	60	

Note: B7-H4^high^ (the combined score>3 points) ; B7-H4^low^ (the combined score≤3 points);

*AFP* α-fetoprotein; *ALT* alanine aminotransferase; *CA19-9* carbohydrate antigen 19-9; *HBsAg* hepatitis B surface antigen; *TNM* tumor lymph node metastasis

^a^ *χ* ^2^-test

^b^ Fisher's exact test

Univariate analysis revealed that microvascular/bile duct invasion, large tumor (> 5 cm), multiple tumor number, poor differentiation, lymphatic metastasis, high TNM stage, and high level of B7-H4 expression were high risk factors for OS and cumulative recurrence (Table 2). Individual clinicopathological features with significance by univariate analysis were adopted as covariates in a multivariate Cox proportional hazards model and then combined variables were further analyzed. Multivariate Cox proportional hazards analysis showed high level of B7-H4 was independent of the other prognostic markers (multiple tumor, lymphatic metastasis, and poor differentiation) for both OS (*p* = 0.001) and cumulative recurrence (Table 2, *p* = 0.002). These data indicate that B7-H4 expression in tumor tissue is a valuable predictor for prognosis of ICC patients.

**Table 2:** Univariate and multivariate analysis of factors associated with OS and cumulative recurrence in 140 ICC patients

Factors	OS		Cumulative recurrence	
	HR (95% CI)	*p*	HR (95% CI)	*p*
*Univariate analysis*				
Age, years (< 53 vs. ≥ 53)	0.865 (0.593–1.262)	0.450	0.821 (0.562–1.199)	0.308
Sex (male vs. female)	0.991 (0.677–1.449)	0.959	0.938 (0.641–1.373)	0.742
HBsAg (negative vs. positive)	0.937 (0.636–1.379)	0.739	0.834 (0.563–1.237)	0.366
Liver cirrhosis(no vs. yes)	0.857 (0.584–1.257)	0.427	0.915 (0.623–1.345)	0.652
Serum AFP* (< 20 vs. ≥ 20, ng/ml)	0.713 (0.405–1.254)	0.241	0.915 (0.511–1.637)	0.764
Serum ALT* (< 75 vs. ≥ 75, μ/l)	1.159 (0.649–2.070)	0.618	0.926 (0.536–1.600)	0.782
Serum CA19-9*(< 37 vs. ≥ 37, ng/ml)	0.841 (0.568–1.246)	0.387	0.817 (0.552–1.211)	0.314
Microvascular/bile duct invasion (yes vs. no)	0.591 (0.367–0.954)	0.031	0.587 (0.363–0.948)	0.030
Tumor size (≤ 5 vs. > 5 cm)	0.560 (0.337–0.929)	0.025	0.485 (0.288–0.816)	0.006
Tumor number (single vs. multiple)	0.421 (0.224–0.793)	0.021	0.355 (0.187–0.674)	0.002
Tumour differentiation (I/II vs. III/IV)	0.676 (0.462–0.987)	0.043	0.667 (0.456–0.975)	0.032
Lymphatic metastasis (no vs. yes)	0.557 (0.364–0.850)	0.007	0.506 (0.334–0.769)	0.001
TNM stage (I/II vs. III/IV)	0.609 (0.407–0.910)	0.015	0.549 (0.369–0.817)	0.003
B7-H4 staining (low vs. high)	0.560 (0.383–0.818)	0.003	0.540 (0.369–0.791)	0.002
*Multivariate analysis*				
Tumor number (single vs. multiple)	0.423 (0.223–0.802)	0.008	0.421 (0.220–0.808)	0.009
Tumor differentiation (I/II vs. III/IV)	0.632 (0.432–0.925)	0.018	0.678 (0.462–0.994)	0.047
Lymphatic metastasis (no vs. yes)	0.541 (0.351–0.833)	0.005	0.525 (0.342–0.806)	0.003
B7-H4 staining (low vs. high)	0.532 (0.363–0.779)	0.001	0.538 (0.366–0.791)	0.002

### High expression of B7-H4 promoted metastasis and invasion of ICC cells in vitro

**Fig. 3:**
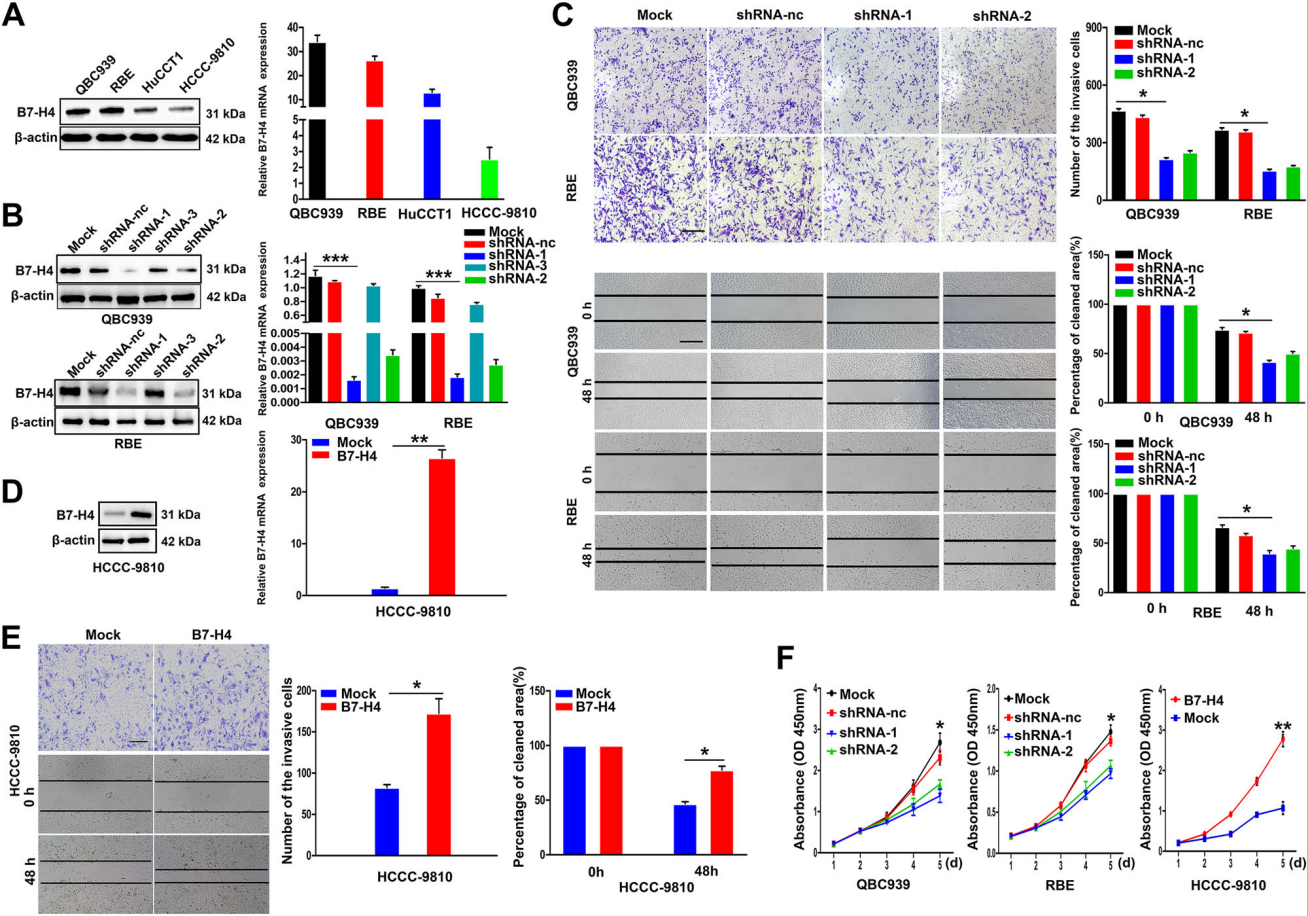
Role of B7-H4 expression in the invasion and metastasis of ICC cells in vitro. **a** Western blotting and PCR assay showed the expression of B7-H4 in four ICC cell lines. **b** Western blotting and qRT-PCR were used to assay the interference efficiency of three sequence of B7-H4 shRNA in QBC939 cells. The sequence 1 and 2 B7-H4 shRNA was validated as high interference efficiency (***p < 0.001) . **c** Transwell  (upper panel) and wound-healing assay (lower panel) showed the inhibited invasion and mobility of ICC cells after the B7-H4 interference (*p < 0.05). **d** Western blotting and qRT-PCR showed upregulated expression of B7-H4 in HCCC-9810 cells transfected with B7-H4 cDNA (**p < 0.01). **e** Transwell (upper panel) and wound-healing assay (lower panel) showed the enhanced invasion and mobility of HCCC-9810 cells transfected with B7-H4 cDNA (×200) (*p < 0.05). **f** Cell proliferation assay showed that ICC cells with high expression of B7-H4 had higher growth rate than those expressing low B7-H4 (*p < 0.05, **p < 0.01)

Here we investigated the role of B7-H4 in ICC cells. We first examined the expression of B7-H4 in four ICC cell lines by western blotting and qRT-PCR, and found that ICC cell line QBC939 and RBE had higher expression of B7-H4 compared with HCCC-9810 and HuCCT1 cells (Fig. 3a). Then, we chose ICC QBC939 and RBE cells with high expression of B7-H4, to construct stably knockdown expression of B7-H4 cells by RNA interference. Short hairpin RNA (shRNA) 1 and 2 target sequence with highly intefered efficiency were chosen for function study (Fig. 3b). Transwell assay showed the impaired invasion of QBC939 and RBE cells after B7-H4 interference (Fig. 3c). Wound-healing assay also showed that knockdown of B7-H4 in QBC939 and RBE cells dramatically inhibited the ability of migration (Fig. 3c). After successful transfection with B7-H4-cDNA in ICC cell line HCCC-9810 with low level of B7-H4 (Fig. 3d), the elevated B7-H4 expression significantly enhanced the ability of invasion and motility of tumor cells (Fig. 3e). We also investigated the role of B7-H4 expression in cell proliferation and found that inhibition of B7-H4 expression in ICC cells resulted in the decreased proliferation rate and vice versa (Fig. 3f). These data revealed that high expression of B7-H4 promoted proliferation, invasion, and migration of ICC cells.

### High expression of B7-H4 promoted tumor growth and progression of ICC cells in vivo

**Fig. 4:**
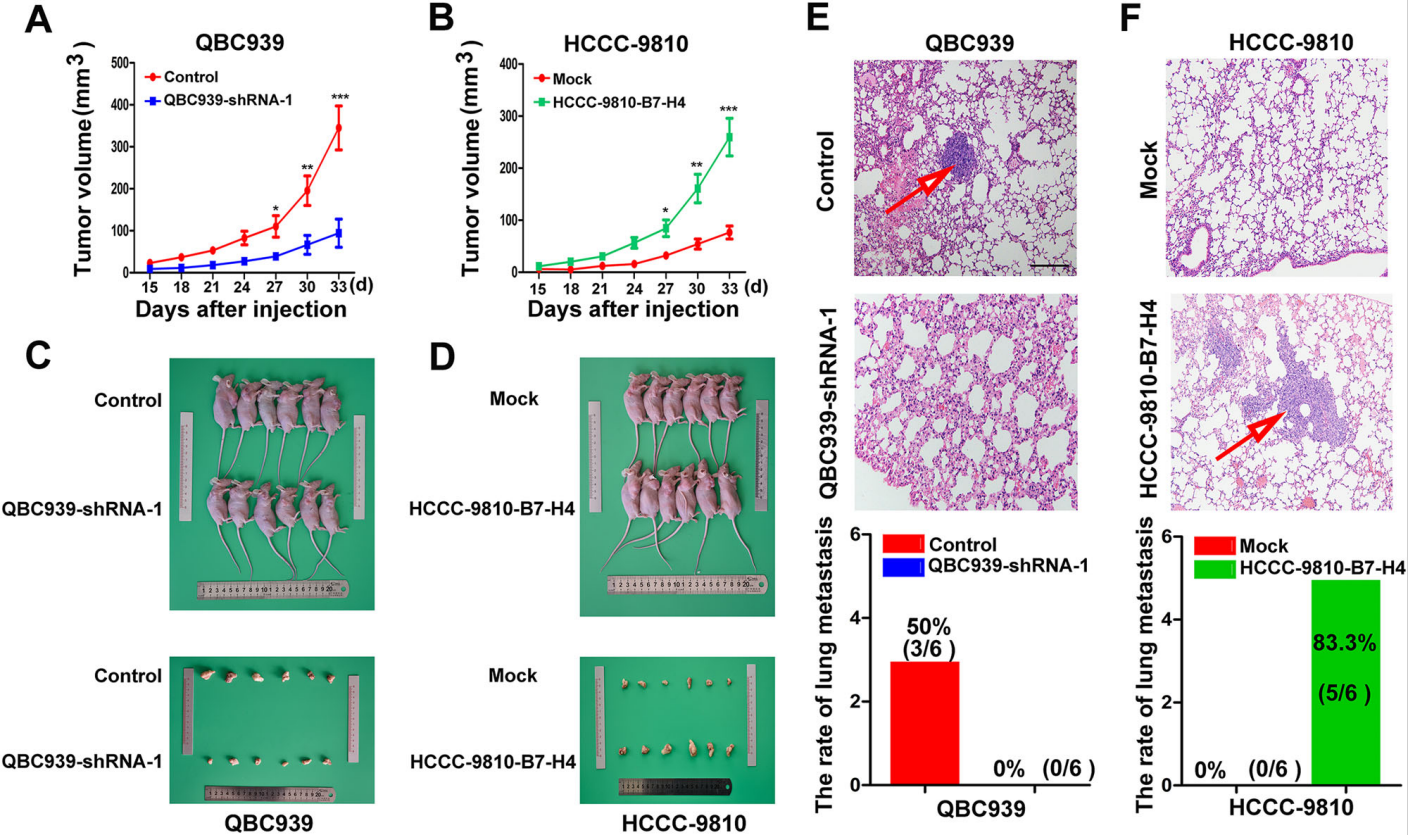
High expression of B7-H4 promoted tumor progression of ICC cells in vivo. **a** Growth curve showed that tumor derived from QBC939-NC cells grew faster than that of QBC939-shB7-H4 group (*n* = 6, *p < 0.05, **p < 0.01, ***p < 0.001). **b** Growth curve showed that tumor derived from HCCC-9810-B7-H4 cells grew faster than that of HCCC-9810-Mock (*n* = 6, *p < 0.05, **p < 0.01, ***p < 0.001). **c** Tumor volume derived from QBC939-NC cells was larger than that of QBC939-shB7-H4 group (*n* = 6). **d** Tumor volume derived from HCCC-9810-B7-H4 cells was larger than that of HCCC-9810-Mock group (*n* = 6). **e** Representative image showed lung metastasis in 50% QBC939-NC cells group, 0 in QBC939-shB7-H4 group. **f** Representative image showed lung metastasis in 83.3% HCCC-9810-B7-H4 cells group, 0 in HCCC-9810-Mock (scale bar = 100 μm)

Next we constructed a subcutaneous xenograft model using QBC939-B7-H4 shRNA cells, HCCC-9810-B7-H4 cells, and their controls. Two weeks after inoculation, all mice successfully formed palpable tumors. Tumor growth curve showed that tumors derived from HCCC-9810-B7-H4 and QBC939-control cells grew faster than those in HCCC-9810-control and QBC939-B7-H4 shRNA cells, respectively (Fig. 4a, b). After innoculation for 33 days, the mice were killed. Tumor volume of HCCC-9810-B7-H4- and QBC939-control-derived xenografts was 256.84 ± 26.64 mm^3^ and 325.66 ± 63.27 mm^3^, which was significantly larger than that derived from HCCC9810-control and QBC939-B7-H4 shRNA groups, respectively (74.35 ± 8.68 mm^3^ and 92.26 ± 12.74 mm^3^, respectively, *p* < 0.001, Fig. 4c, d). Then, we investigated the lung metastasis rate using serial section and found that the pulmonary metastasis rate was 50% (3/6) in the QBC939-control group, 83.3% (5/6) in the HCCC-9810-B7-H4 group, and 0% (0/6) in both QBC939-B7-H4 shRNA and HCCC9810-control group (*p* < 0.05) (Fig. 4e, f). Collectively, these results indicate that B7-H4 could significantly promote tumor growth and tumor progression of ICC cells in vivo.

### High B7-H4 expression promoted tumor progression of ICC cells through induction of EMT, inhibition of appotosis, and activation of ERK1/2 signal

**Fig. 5:**
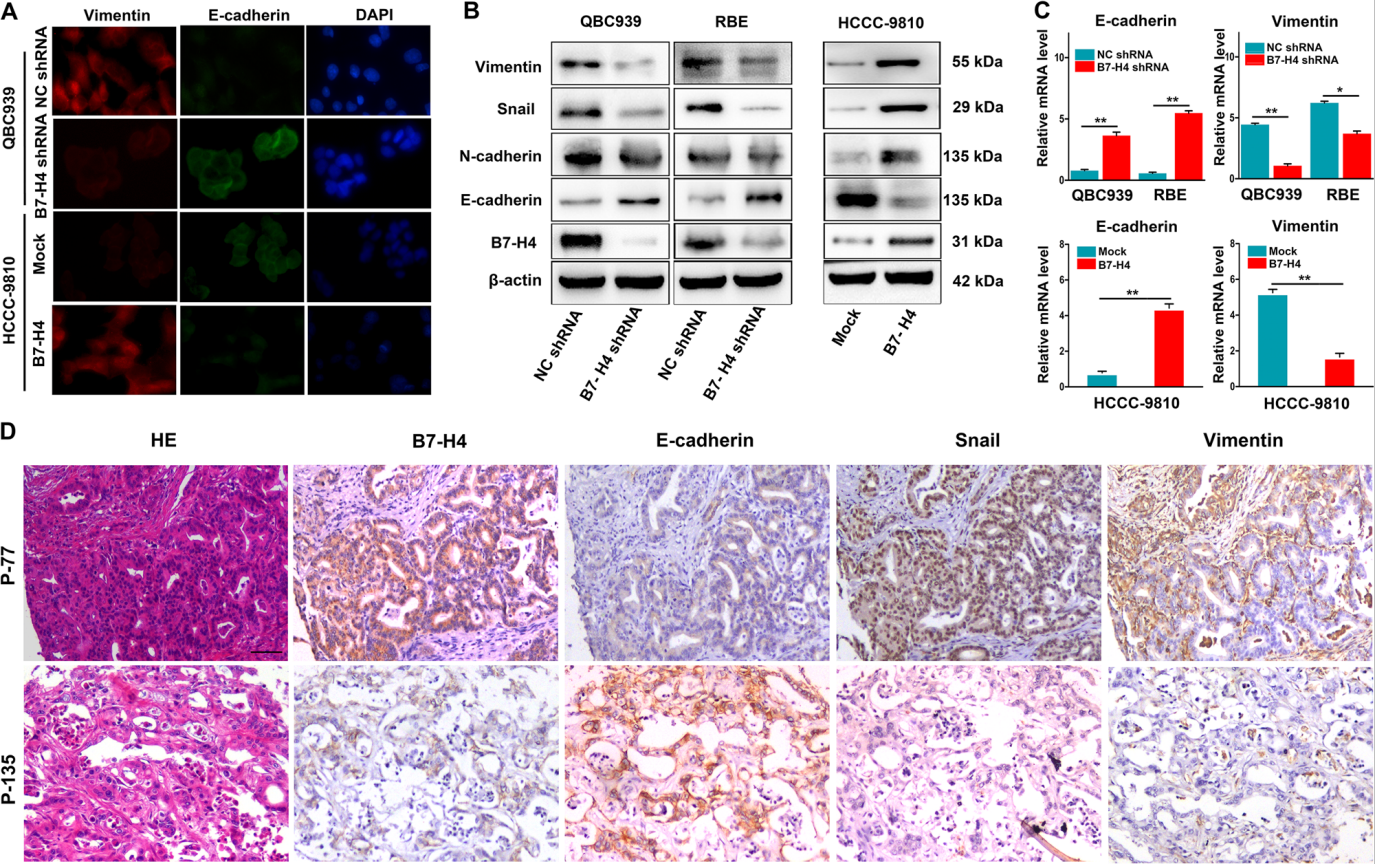
The relationship between expression of B7-H4 and the expression of EMT-related marker in ICC. **a** Immunofluorescence showed that Vimentin expression was downregulated and E-cadherin was upregulated in QBC939 interferenced with B7-H4, whereas Vimentin was upregulated and E-cadherin was downregulated after HCCC-9810 transfected with B7-H4 cDNA. **b** Western blotting showed the expression of Vimentin, snail, N-cadherin, and E-cadherin in ICC cells modified with the expression of B7-H4. **c** qRT-PCR showed the expression of E-cadherin mRNA and Vimentin mRNA in ICC cells modified with B7-H4 expression. *p < 0.05, **p < 0.01. **d** Serial section and IHC staining showed ICC tissue with high expression of B7-H4 had tendency to have weak expression of E-cadherin and strong staining of Vimentin and snail. Scale bar = 200 μm

Recent studies have showed that tumor cells mainly undergo epithelial-to-mesenchymal transitions (EMT) to acquire the ability of motility and invasiveness^18,19^. Thus, we performed immunofluorescence and western blotting, to determine the expression of EMT-related markers in ICC cells with different level of B7-H4. Immunofluorescence (IF) assay showed that down-regulation of B7-H4 in ICC cellls resulted in the increased expression of epithelial marker E-cadherin and the decreased expression of mesenchymal marker Vimentin (Fig. 5a). Western blotting showed a downregulated expression of Snail, Vimentin, and N-cadherin, and an upregulated expression of E-cadherin in ICC cells after interference of B7-H4 expression, vice verse (Fig. 5b). qRT-PCR also validated that E-cadherin mRNA was upregulated in ICC cells after interference with B7-H4, whereas Vimentin mRNA was significantly inhibited (Fig. 5c). We further used the IHC to investigate the relationship between B7-H4 expression and expression of EMT-related markers in the 140 ICC tissues. The results showed that tumor sample expressing high B7-H4 tended to have up-regulation of Vimentin and snail and downregulation of E-cadherin (Fig. 5d).

**Fig. 6:**
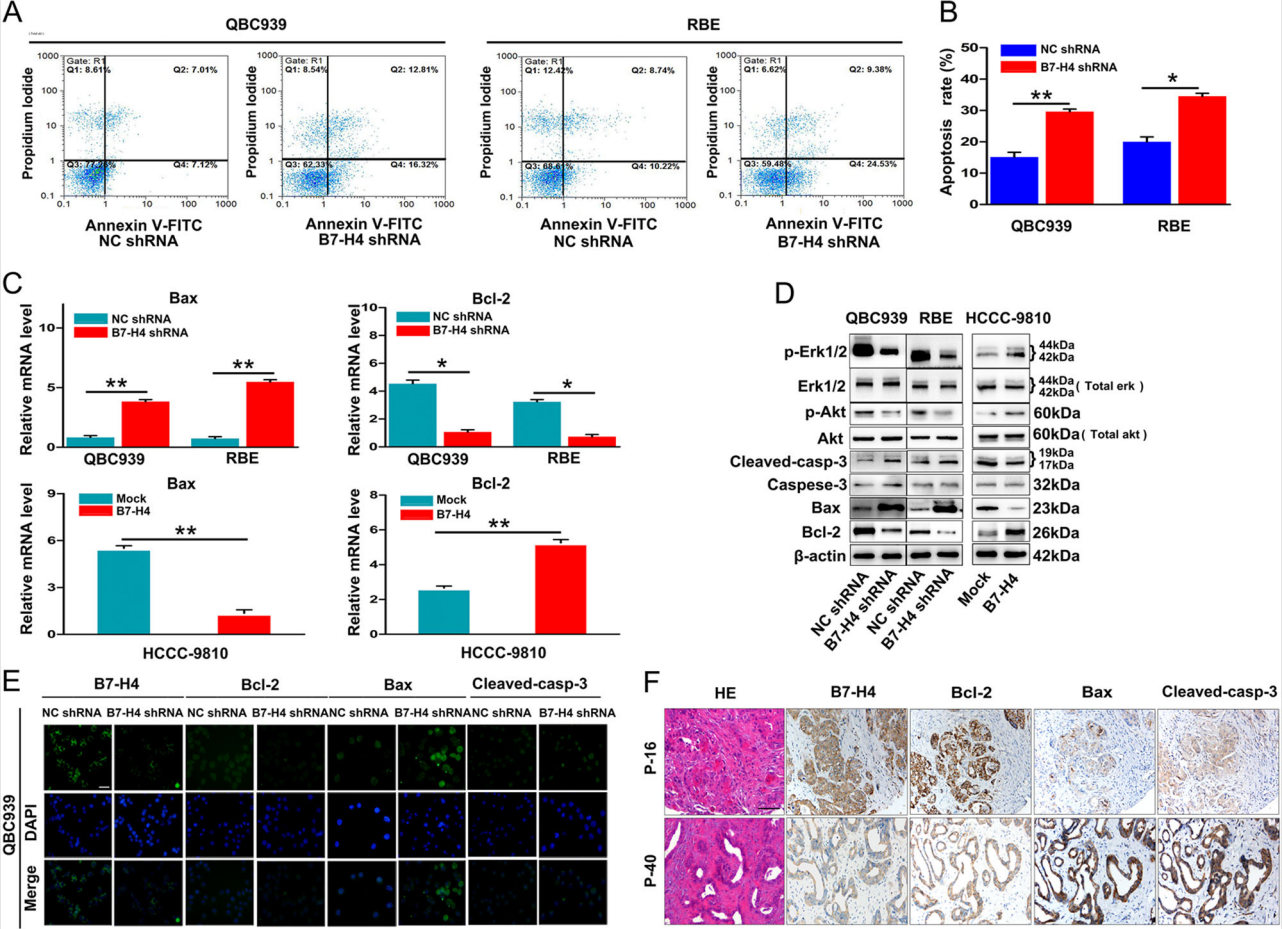
High expression of B7-H4 in ICC activated the ERK1/2 and inhibited apoptosis. **a**,** b** Flow cytometry showed increased apoptosis rate in ICC cells interference with B7-H4 (*p < 0.05, **p < 0.01). **c** qRT-PCR showed that upregulation of B7-H4 expression in ICC cells resulted in increased expression of bcl-2 mRNA and decreased expression of Bax mRNA(*p < 0.05, **p<0.01). **d** Western blotting showed that the expression of Bcl-2 and p-Erk1/2 in QBC939 and RBE cells were downregulated after B7-H4 interference, whereas Bax, cleaved-Caspase-3 expression were upregulated. The opposite results were observed in ICC cells transfected with B7-H4 cDNA compared with the HCCC-9810-control cells. **e** Immunofluorescence analysis showed that Bcl-2 expression was downregulated and the expression of Bax and cleaved-caspase-3 were upregulated in QBC939 cells interferenced by B7-H4. **f** Representative images showed that ICC tumor samples with B7-H4^high^ had tendency to have high expression of Bcl-2 and low Bax expression, whereas ICC tumor samples with B7-H4^low^ presented low expression of Bcl-2 and high expression of Bax and cleaved-caspase-3. Scale bar = 200 μm

Recent studies showed that B7-H4 is also involved in the apoptosis^20,21^. In present study, we also determined the role of B7-H4 expression in apoptosis of ICC cells. Unexpectedly, we found an increased apoptosis rate in QBC939 and RBE cells after B7-H4 interference by flow cytometric analysis (Fig. 6a, b). Moreover, an increased expression of Bax mRNA and a decreased expression of Bcl-2 mRNA were found in ICC cells after B7-H4 interference. Interestingly, Bcl-2 mRNA was increased and Bax mRNA was decreased after expression of B7-H4 in ICC cells was forced (Fig. 6c). Then, western blotting also validated above results (Fig. 6d). In addition, increased expression of cleaved-Caspase-3 and decreased expression of ERK1/2 phosphorylation were detected in ICC cells transfected with B7-H4 interference. Accordingly, the level of ERK1/2 phosphorylation were upregulated in HCCC-9810 cells transfected by B7-H4 complementary DNA (Fig. 6d). IF also showed the same change of Bcl-2, Bax, and cleaved-Caspase-3 expression in ICC cells with B7-H4 interference (Fig. 6e). Interestingly, IHC staining also showed that ICC patients with high level of B7-H4 had tendency to high level of Bcl-2 and low level of Bax and cleaved-Caspase-3 (Fig. 6f). These results indicate that high levels of B7-H4 probably promote tumor progression of ICC cells through induction of EMT, inhibition of apoptosis, and activation of ERK1/2 signal.

## Discussion

In the present study, our data showed that B7-H4 expression was overexpressed in ICC tissues. Inhibition of B7-H4 expression impaired invasion and accelerated apoptosis of ICC cells. Importantly, inhibition of B7-H4 expression in ICC cells markedly impaired tumor growth and lung metastasis in vivo. Clinically, high expression of B7-H4 in tumor tissues positively correlated to malignant phenotype and the poor prognosis of ICC patients. In the present study, we also detected that B7-H4-positive tumor-associated macrophages and other immune cells in tumor tissues, but their numbers were less than those in correspondingly adjacent normal tissues (unpublished data). Although we did not completely exclude immune cells expressing B7-H4 from tumor progression of ICC, above results provide enough evidence to support that B7-H4 have a substantial role in the progression of ICC.

It is well known that malignant tumor cells usually undergo the EMT process to gain the invasive and metastatic properties.^18,19^ Our data showed that ICC cells with high expression of B7-H4 presented with high ability of metastasis and invasion. Moreover, in vitro experiment showed that elevated expression of B7-H4 in ICC cells could upregulate the expression of mesenchymal marker Snail and Vimentin, and downregulated the expression of epithelial marker E-cadherin. In serial section of tumor samples from ICC patients, the high level of B7-H4 in tumor tissues were positively consistent with loss of E-cadherin and strong staining for Vimentin and Snail. Furthermore, high level of B7-H4 endowed ICC cells with the ability of apoptosis-resistance, which indicate that tumor cells undergoing EMT process can override oncogene-induced premature senescence and apoptosis. In addition, we further showed that high level of B7-H4 was positively associated the activation of mitogen-ctivated protein kinase and phosphoinositol 3-kinase signaling, which may be an indirect effect of B7-H4. Thus, our results definitely suggest that the high level of B7-H4 in ICC cells promote tumor progression through EMT of tumor cells.

Recently, several studies reported that B7-H4 had an oncogenic role in multiple tumor types and high level of B7-H4 could serve as a biomarker for predicting poor prognosis of cancer patients.^12,15^ In the present study, our results also showed that ICC patients with high expression of B7-H4 in tumor tissues had poor prognosis. Moreover, high expression of B7-H4 was significantly related to malignant phenotype, such as lymph node metastasis, high TNM stage, and poor tumor differentiation. Inhibition of B7-H4 expression in ICC cells showed better control of tumor growth in mice. Although we lack direct evidece of serum B7-H4 for predicting prognosis of ICC patients, we believe that B7-H4 may appear as a novel marker in predicting the prognosis and a potential therapeutic target for those patients.

## Materials and Methods

### Patients and specimens

The tumor and adjacent non-tumor samples including 35 freshly frozen tissues and 140 formalin-fixed paraffin-embedded tissues were continuously obtained from ICC patients who underwent a curative resection between 1999 and 2006 at the Liver Cancer Institute of Zhongshan Hospital, Fudan University (Shanghai, China). The histopathological diagnosis of ICC was based on World Health Organization criteria. Follow-up data were collected until February 2009 and the median duration of follow-up was 25 months (range 4–120 months). Written informed consent was obtained from each patient. This study were approval by the Zhongshan Hospital Research Ethics Committee.

### Tissue microarrays and immunohistochemistry

The construction of the TMAs was performed as described in our earlier study^18,19,22,23^. Briefly, 140 pairs of ICC tissues and adjacent non-tumoral tissues were stained with hematoxylin and eosin (H&E), and diagnosed by two pathologists. Representative areas away from necrotic and hemorrhagic loci were chose to take two biopsies of 1 mm in diameter from paraffin blocks and then transferred to the defined array positions. Four-millimeter-thick serial sections were taken on 3-aminopropyltriethoxysilane–coated slides (Shanghai Biochip Co., Ltd). IHC was performed using a two-step protocol as previously described^19^. Briefly, paraffin sections were baked for 30 min at 70 ℃, de-paraffinized in xylene, rehydrated in gradually varied alcohol, and then the sections were managed with 1% H_2_O_2_ to neutralize endogenous peroxidase for 30 min. The antigen retrieval was processed with incitrate buffer (pH 6.0) in a microwave oven. After antigen retrieval, the sections were incubated with primary antibody and secondary antibody. Omission of primary antibody was set as negative control. The antibodies used for IHC was listed in Supplementary Table S1. The sections were then stained with DAB (3,3-diaminobenzidine) and terminated in phosphate-buffered saline (PBS), and then counterstained with hematoxylin. Images were photographed by Leica Q Win Plus v3 software (Leica Microsystems Imaging Solutions, Cambridge). The B7-H4 was immunohistochemically stained yellow or brown positioning in the cell membrane. The samples were scored independently according to the intensity of cellular staining and the proportion of stained cells. The proportion of stained cells in total cell number of every point about 0, 1–25%, 25–50%, 50–75%, and > 75% were scored as 0, 1, 2, 3, and 4 points, respectively. The intensity of cellular staining was determined by the degree of color, namely none, weak, medium, and strong were scored as 0, 1, 2, and 3 points, respectively. The final combined score 0–7 point for each patient was decided and defined the score ≤ 3 points as the B7-H4^low^ group and the score > 3 points as the B7-H4^high^ group. The intensity of E-cadherin, Vimentin, snail, Bcl-2, Bax, and Cleaved-casp-3 were evaluated as previously described.^18,22–24^

### Cell culture

The human ICC cell line QBC939 was provided by the Shanghai Cancer Institute (Shanghai, China), and RBE, HCCC-9810, and HuCCT1 cell lines were purchased from the Chinese Academy of Sciences Shanghai Branch Cell Bank (Shanghai, China). Cell lines were cultured in RPMI Medium 1640 (Gibco, USA) supplemented with 10% fetal calf serum (Gibco), penicillin (100 units/ml), and streptomycin (100 µg/ml) at 37 ℃ in a thermostatic incubator with 5% CO_2_.

### Western blotting

Western blotting was done as described in our earlier study^23,25^. Total protein from ICC cells and tumor specimens from 35 ICC patients were extracted and separated in 10% SDS-PAGE and then electro-transferred onto polyvinylidene difluoride membranes. After blocked in Blocking Buffer (Beyotime, China) for 1 h, the membranes were incubated with primary antibody and secondary antibody according to protocols of the manufacturer. The relative expression of B7-H4 was analyzed by the comparative β-actin using gray level difference analysis as previously described.^21^ Primary antibodies were listed in Supplementary Table S1. All experiments were performed in triplicate.

### RNA extraction and real-time PCR

ICC cells and frozen tumor specimens from 35 ICC patients were used to assay the expression of B7-H4 mRNA. qRT-PCR was carried out as described previously^22,23^. Breifly, total RNA was extracted using the Trizol reagent (Invitrogen) and reverse transcribed using RevertAid first-strand cDNA synthesis kit (Fermentas). Glyceraldehyde 3-phosphate dehydrogenase (GAPDH) was used as an internal control. Primer sequences of B7-H4 and GAPDH were listed in Table 3. Amplification and detection were performed using the ABI PRISM 7900 Sequence Detection System (Applied Biosystems, Foster City, CA). The relative expression of B7-H4 was analyzed by the comparative cycle threshold (Ct) method. All experiments were performed in triplicate.

**Table 3:** qRT-PCR primer and vshRNA sequences

Gene	Sequence
*B7-H4 shRNA*	
B7-H4 vshRNA1	5′-GGATATCAAAGTGACAGAATC-3′
B7-H4 vshRNA2	5′-GGAAGTGAATGTGGACTATAA-3′
B7-H4 vshRNA3	5′-GAACCTGACATCAAACTTTCT-3′
*B7-H4 primer*	
Forward	5′-CATCATCATTATTCTGGCTGGAG-3′
Reverse	5′-AGGCGACAGTAGTGACTGTGATGG-3′
*GAPDH primer*	
Forward	5′-TGCCAA ATATGACATCAAGAA-3′
Reverse	5′-GGAGTGGGTGTCGTCGCTGTTG-3′
*Bcl-2 primer*	
Forward	5′-TACGAGTGGGATACTGGAGATGAA-3′
Reverse	5′-TCAGGCTGGAAGGAGAAGATG-3′
*Bax primer*	
Forward	5′-CAGGACGAGTCCACCAAGAAG-3′
Reverse	5′-GCAAAGTAAAACAGGGCGACA-3′
*E-cadherin primer*	
Forward	5′-GGCCAGCCATGGGCCCTTGG-3′
Reverse	5′-CACCTTCAGCCAACCTGTTT-3′
*Vimentin primer*	
Forward	5′-CTTCGCCAACTACATCGACA-3′
Reverse	5′-GCTTCAACGGCAAAGTTCTC-3′

*qRT-PCR.* quantitative real-time PCR

### IF and flow cytometry assay for apoptosis

IF was used to investigate the expression of Vimentin, E-cadherin, Bax, and Bcl-2 in ICC cells as described previously.^22^ Apoptosis were assayed as previously described.^26^ Cells were fixed in 70% ethanol, cellular DNA was stained with propidium iodide (Sigma 81845), and analyzed by flow cytometer (Becton Dickinson, Franklin Lakes, NJ).

### Gene silencing and transfection

Three different sequences targeted to different sites in B7-H4 mRNA were designed and provided by Ribobio Company (Guangzhou, Guangdong, China). The sense and antisense strands of shRNA are shown in Table 3. The B7-H4 cDNA lentiviral vector and B7-H4- shRNA expression lentivirus were constructed (Shanghai GeneChemCo.) as described previously.^27^ Stably transfectant clones were characterized using qRT-PCR and western blotting for their expression levels of B7-H4.

### Cell proliferation assay

Cell proliferation assay was performed as the manufacturer’s instructions of Cell Counting Kit-8 assay (KeyGEN Biotech, Nanking, China). One thousand cells were seeded in 96-well plates. After incubation with the CCK-8 reagent (2-(2-methoxy-4-nitrophenyl)-3-(4-nitrophenyl)-5-(2,4-disulfophenyl)- 2H-tetrazolium monosodium salt) for 3 h, absorbance at 450 nm was measured using Infinite M200 (Tecan, Switzerland). Each sample was assessed in triplicate at 0 day, first, second, thirth, fourth, and fifth day.

### Transwell  invasion and wound-healing assay

Transwell invasion was performed as described previously with slight modification^18,27^ . Briefly, 1 × 10^5^ ICC cells were suspended in Dulbecco’s modified Eagle’s medium (DMEM) without fetal bovine serum and added to the upper chamber precoated with matrigel (BD Biosciences, San Jose, CA, USA). The supernatant of NIH3T3 in DMEM with 10% fetal bovine serum was added in the lower chamber as chemoattractant. The cells were incubated for 24 h at 37 ℃ in 5% CO_2_ and fixed with 4% paraformaldehyde, stained with 0.1% crystal violet (Sigma, St. Louis, MO, USA), and enumerated the invased cells in five randomly selected areas under a light microscope (×200). All assays were performed in triplicate.

Wound-healing assay was performed as described previously^23,25^. Briefly, 1 × 10^6^ ICC cells cells were cultured in 6-well plates and allowed to form a confluent monolayer for 24 h. The cells were starved in serum-free DMEM, scratched using pipette tips, washed with PBS, and photographed using a phase-contrast microscope for 0, 24, and 48 h. The migrated cells were subsequently quantified via manual counting and the inhibition ratio was expressed as a percentage of the control. All assays were performed in triplicate.

### In vivo tumor growth and metastasis assay

Four-week-old male nude mice were purchased from Kunming Medical University Experimental Animal Center (Kunming, China) and raised according to the guidelines provided by Kunming Medical Experimental Animal Care Commission. ICC cells (5 × 10^6^) were used to establish subcutaneous (SC) xenograft tumor model as previously described^27^. Tumor growth was monitored each 3 days and mice were killed after innoculation 33 days. Two dimensions of tumors were measured as previously described^27^. Serial section of each lung was stained by H&E and metastatic rate was assayed as previously described^24^.

### Statistical analysis

The statistical analyses were conducted using SPSS 17.0 software (SPSS, Chicago, IL) and PRISM 5.0 (GraphPad Software Inc., San Diego, CA, USA) as previously described^18,23,24^. Values are expressed as the mean ± SD. For comparisons of individual variables, Student *t*-tests, *χ* ^2^-tests, and Spearman's coefficient tests were used. *p* < 0.05 was considered statistically significant. OS was calculated by the Kaplan–Meier method and was analyzed by the log-rank test. A Cox proportional hazards regression model was used to analyze independent prognostic factor. *p* < 0.05 was considered statistically significant.

## Electronic supplementary material

Supplementary Table 1
