# Supplementary material for: Upregulation of B7-H4 promotes tumor progression of intrahepatic cholangiocarcinoma
Source: Cell Death Dis. 2017 Dec 13;8(12):3205. doi: 10.1038/s41419-017-0015-6 (PMC5870586; doi:10.1038/s41419-017-0015-6)
Supplement: Supplementary file 1 — Supplementary Table 1 [file 41419_2017_15_MOESM1_ESM.docx]

**Supplementary Table 1** **List of primary antibodies used in the study**

| Antibody | Applications | Company |
| --- | --- | --- |
| B7-H4 | WB, IHC, IF | Abcam (NO.ab209242) |
| Snail  Vimentin | WB, IHC  WB, IHC, IF | Cell Signaling Technology  (NO.#3879)  Abcam (NO.ab8978) |
| N-cadherin | WB | Abcam (NO.ab18203) |
| E-cadherin | WB, IHC, IF | Abcam (NO.ab1416) |
| β-actin | WB | Sigma (NO.SAB5500001) |
| p-Erk1/2 | WB | Abcam (NO.ab151279) |
| Erk1/2 | WB | Cell Signaling Technology  (NO.#9194S) |
| p-Akt | WB | Abcam (NO.ab151279) |
| Akt | WB | Abcam (NO.ab151279) |
| Cleaved caspase-3 | WB, IHC, IF | Abcam (NO.ab136812) |
| Caspase-3 | WB | Abcam (NO.ab13586) |
| Bax | WB, IHC, IF | Abcam (NO.ab79217) |
| Bcl-2 | WB, IHC, IF | Abcam (NO.ab194583) |

Abbreviations: WB, western blot; IHC, immunohistochemistry;

IF, immunofluorescence
